# Towards an Understanding of Conservation-Based Costs, Benefits, and Attitudes of Local People Living Adjacent to Save Valley Conservancy, Zimbabwe

**DOI:** 10.1155/2018/6741439

**Published:** 2018-09-02

**Authors:** Given Matseketsa, Gladman Chibememe, Never Muboko, Edson Gandiwa, Kudakwashe Takarinda

**Affiliations:** School of Wildlife, Ecology and Conservation, Chinhoyi University of Technology, Private Bag 7724, Chinhoyi, Zimbabwe

## Abstract

Communities juxtaposed to protected areas (PAs) often disproportionally accrue the costs of conservation, but they can also receive benefits from the existence of a PA. The extent to which local communities benefit or incur costs as a result of residing next to PAs is of interest to conservationists and policy-makers. This study sought to understand the costs, benefits, and attitudes of local people living adjacent to Save Valley Conservancy (SVC), Zimbabwe. The purpose was to determine whether benefit and loss accrual has a bearing on the levels of illicit wildlife-based activities experienced in the SVC. Data were collected through a household questionnaire survey and key informant interviews from April to July 2014. A three-stage sampling was adopted: firstly, purposive sampling was employed to select wards adjacent to the SVC; secondly, random sampling was used to select villages within the selected wards; and thirdly, systematic sampling was used to select 71 household questionnaire respondents. Snowball sampling was used to select 9 key informants. The study results show that the majority of locals living close to SVC are not deriving discernable benefits and the costs of conservation are escalating influencing negative attitudes towards wildlife conservation, thus causing them to view wildlife as a nuisance. Overall, our results indicate that conservation losses and benefit accrual by local communities influence their attitudes toward SVC and conservation in general. We conclude that costs incurred outweighed the benefits accrued, a situation that triggers a more negative form of reciprocity towards SVC and wildlife conservation. It is recommended that a more socially and economically inclusive management approach based on a stakeholder-driven access and benefit sharing (ABS) framework be instituted to promote a more positive form of reciprocity towards SVC and nature conservation.

## 1. Introduction

The advent of colonialism in the 18th and 19th centuries in Africa saw the twin processes of land and wildlife alienation creating hostility to wildlife among the affected local people. Colonialism was the entry point through which the fortress conservation doctrine slithered its way into Africa [1, 2]. This mode of conservation spearheaded human-nature dichotomy through the conceptualization of native resource users as the conservation problem [3–5]. So Africans apart from being ignored, were overwhelmed, manipulated, and outmaneuvered by a conservation crusade led, orchestrated, and dominated by white settlers. Above all, control over natural resources was wrested from them, and livelihood practices such as traditional hunting got criminalized [3, 6, 7]. The colonizers became avid gamekeepers and the Africans the poachers. Thus, the rural poor had to suffer the consequences of living with wildlife while reaping no benefits from wildlife conservation [8, 9]. As a result, one of the dominant conclusions that may be drawn from the decades of research on the social dynamics of biodiversity conservation is that protected areas (PAs) have added hardship to households in rural communities throughout much of the African countries [10]. Consequently, PAs in Africa share common salient features: historical poor public relations and minimal support from local communities [11, 12]. The land where the natives once hunted game both for food and ritual became enclosed and privatized and what was once an everyday practice became illicit overnight [8, 13, 14]. Hence, PAs have been heavily criticized for preserving nature for a wealthy elite [15]. Thus, this ethnocentric conservation strategy characterized with exclusion has not gained acceptance, as it works against the economic and social interests of local people, and frequently transformed wildlife from an asset into a threat and nuisance [16–18]. Scherl et al. [19] posit that PAs should not exist as islands, divorced from the social, cultural, and economic context in which they are located. Thus, PAs in most developing and independent countries have paid attention to the issue of communities deriving benefits from them and they have made this phenomenon become a more practical and ethical necessity: practical, because to survive, PAs in the poorer nations must be seen as a land use that contributes as positively to sustainable development as the other types of land-use, and ethical, because human rights and aspirations need to be assimilated into national and global conservation strategies if social justice is to be realized [19].

In recent years, after a period of strictly centralized wildlife management and exclusive wildlife conservation, there has been a commendable attempt to balance the needs for conservation with those for rural development [20]. In an effort to redress the colonial injustices, well-meaning conservationists have embraced the paradigmatic shift in the conservation of wildlife from the historical separatist conservation approaches termed “conservation against the people” by Baldus [8] to present day community-based natural resources management (CBNRM) [20]. Thus, the modern movement in conservation now recognizes PAs to be socioecological systems as it has been proven beyond doubt that no PA can succeed for long in the teeth of local opposition [21]. Beyond these advancements, narrowing down to the Zimbabwean case, political independence has championed the resurgence of restoring the right to own and manage wildlife that had been denied in the colonial era to rural communities. In this regard, the state pioneered the Communal Areas Management Programme for Indigenous Resources (CAMPFIRE) initiative [20]. However, the extent to which local communities outside the CAMPFIRE-based projects benefit or incur losses due to conservation efforts in general is still a mystery. The objectives of this study were therefore to (i) establish the nature of costs incurred by communities living adjacent to the SVC, (ii) determine the benefits local communities derive from SVC, and (iii) assess local communities' attitudes towards the SVC.

### 1.1. Conceptual Background

**Figure 1:**
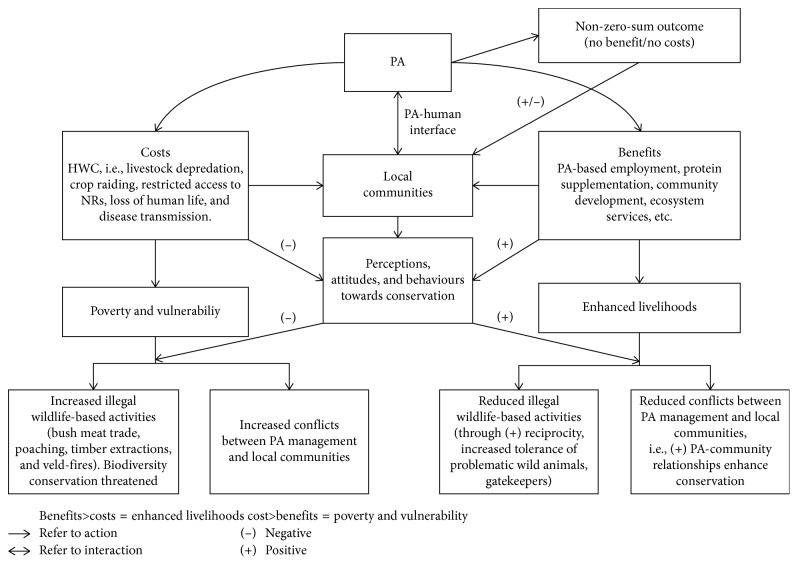
Conceptual framework denoting reciprocity in PA-local community interface based on the social exchange theory (SET). PA, protected area.

The conceptual framework (Figure 1) explains the interaction occurring between PA management and local communities. The conceptual framework is premised on the following four assumptions:

- Local communities seek benefits and avoid costs
- Local communities are rationale beings
- The standards that local communities use to evaluate costs and benefits vary over time from person to person
- Relationships are interdependent and dynamic

The proposed framework examines the relationship between the PA management and the local communities who are either positively or negatively affected by the PA. Two factors that influence PA-community relationships are identified and each of these factors affects the way PA management and communities relate with each other. Costs and benefits as outcomes of conservation affect the way PA management and communities relate with each other. If communities do not receive benefits and bear costs, they are likely to have a negative relationship with the PA [22, 23]. If the PA management also do not see the importance of extending some benefits to the communities or minimizing levels of wildlife depredation on people's crops and livestock, they are likely to have a negative relationship with the communities. In an attempt to provide a fresh perspective of assessing PA-community relationships, the framework was developed from the social exchange theory (SET) which was propounded by Homans [24]. The social exchange theory dictates that rational human beings base their behavioral choices on maximizing gains and minimizing costs [25], implying that if local people benefit from the existence of a PA, they will be more likely to support conservation and the continued existence of the PA.

## 2. Materials and Methods

### 2.1. Study Area

**Figure 2:**
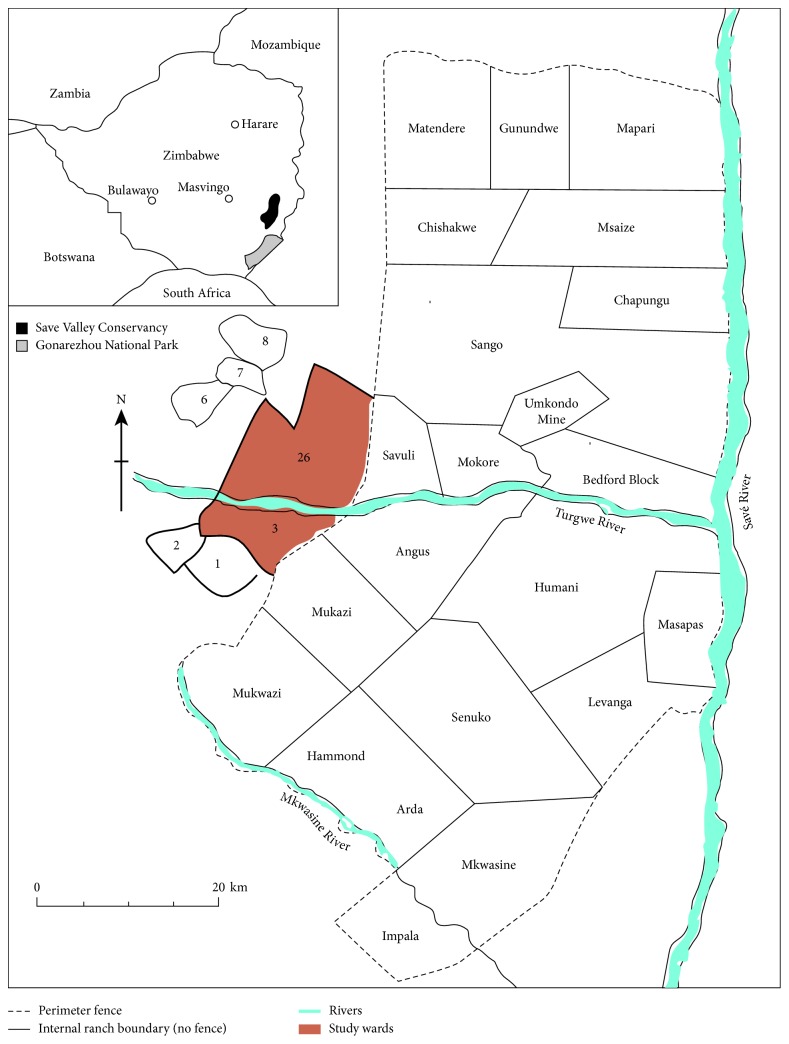
Location of the study wards 3 and 26 adjacent to the southwestern SVC, Zimbabwe.

The study was conducted in two wards: Ward (3) and Ward (26) in Bikita district adjacent to the southwestern border of SVC, southeastern Zimbabwe (Figure 2). SVC spans an area of 3 400 km^2^. Up until April 2014, it was a cooperatively managed private wildlife area, but in the month of May 2014, it was placed under the custodianship of the Zimbabwe Parks and Wildlife Management Authority (ZPWMA). The conservancy is located in agroecological region V which is a semiarid area in the South East Lowveld of Zimbabwe. Its southern boundary is approximately 45 km northeast of Chiredzi town, whereas the Save River and Sangwe communal lands mark its eastern boundary. Its northern boundary lies not far from Birchenough Bridge, and its western boundary is formed by a resettlement scheme on land of the former Devuli Ranch and to the South by Matsai Communal area. It is found in the province of Masvingo and covers two districts, which are Chiredzi and Bikita. It is surrounded by three other districts, which are Zaka, Buhera, and Chipinge. The conservancy is bordered primarily by high-density communal land (of between 11 and 82 people per km^2^) [26], with some commercial agriculture to the south and east.

### 2.2. Data Collection

A mixed-methods approach was used in data collection; it comprised structured household questionnaires which were administered via in-person interviews; the questionnaires included both open-ended and fixed response questions. Fixed response questions were used to ensure precision of responses, whilst open-ended questions were also included to obtain more detailed information on the nature of costs and benefits of conservation. The researchers also used participatory rural appraisal techniques particularly making use of semistructured interviews with key informants. The semistructured interviews were used to solicit more information. Schensul et al. [27] noted that semistructured interviews enable respondents to provide more elaborate and complete answers than fully structured questionnaires, and are flexible enough to allow people to explain their views in their own words, which can be valuable in terms of truly understanding the nature of a particular situation.

In addition, in order to achieve successful field data collection, a three-stage sampling design was adopted due to the nature of the sampling frame, to select the sampling units. The first stage was purposive sampling, where two wards (3 and 26) were selected from a total of eight wards which were under consideration for sampling. Purposive sampling was found suitable as these study sites were under the PA-community outreach initiative and also due to their geographical proximity to the SVC boundary. The second sampling stage used was simple random sampling for village selection where five villages from eleven villages constituting the two wards were selected (i.e., Matsai (78), Village 2 Angus (65), twenty-six (73), twenty-seven (65), and thirty-one (69) with a total of 350 households). The third sampling technique, systematic sampling, was done to select 71 households where questionnaires were administered and collected in the 5 randomly selected villages representing 20% of the targeted population; this was ensured by walking through the village interviewing every second household. In addition, key informants interview questionnaire guides were administered to nine community leaders including traditional leaders, village elders, and ward councilors, all from the abovementioned wards through snowball sampling.

### 2.3. Data Analysis

Descriptive statistics were used to summarize quantitative data sets from household questionnaires. A nonparametric (Kruskal–Wallis-chi-square (*χ*^2^)) test was also used to determine whether given responses on costs, benefit from and attitudes towards the SVC differ across the five villages. Quantitative analyses were conducted using Statistical Package for Social Sciences version 19 for Windows (IBM SPSS Inc., Chicago, USA). Qualitative data from semistructured interviews with key informants were summarized into percentages, and a qualitative content analysis technique was used.

## 3. Results

### 3.1. Responses from Questionnaire Interviewees on Costs Associated with Living Closer to SVC

#### 3.1.1. Claims to Incur Wildlife-Induced Costs from SVC

**Figure 3:**
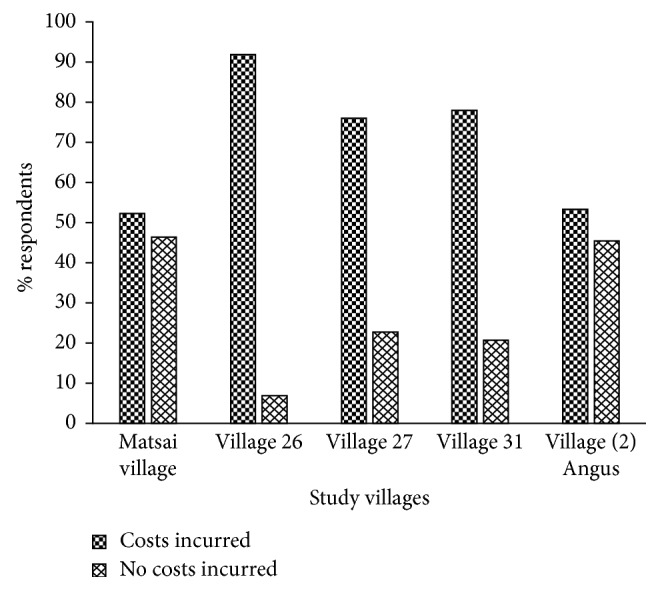
Claims of wildlife-induced costs by local communities across the villages.

In village twenty-six, a high proportion of respondents (*n*=14; 93%) claimed that they incurred costs from wildlife whilst a minor proportion of the respondents (*n*=1; 7%) claimed no such costs (Figure 3). There was no significant difference (KW*χ*^2^ = 9.276, df = 4, *p* > 0.05) in respondents claims for incurring costs from wildlife across villages.

### 3.2. Nature of Costs Incurred

**Figure 4:**
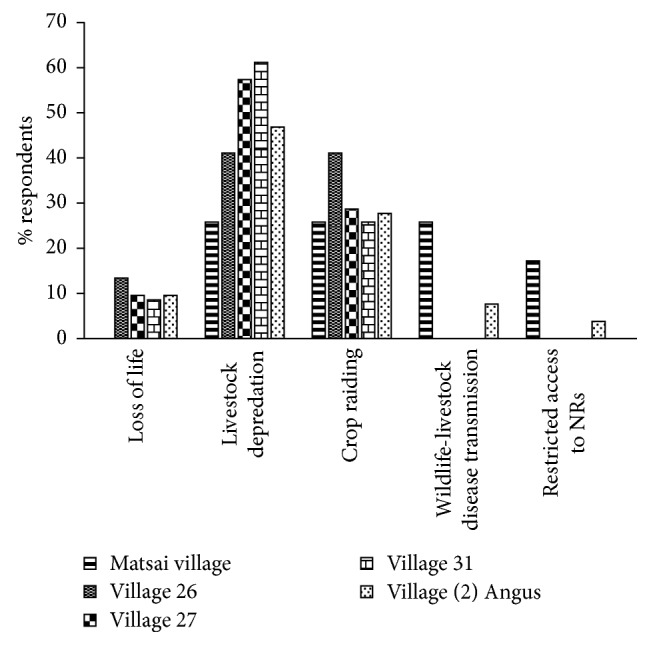
Nature of costs incurred by local communities across the villages.

Respondents incur different kinds of costs from conservation in the SVC. Most (*n*=7; 64%) of the respondents in village thirty-one reported livestock depredation to be the most prevalent cost incurred whilst a few (*n*=2; 18%) of the respondents in village (2) Angus mentioned restricted access to natural resources as a cost incurred (Figure 4). There was a significant difference (KW*χ*^2^ = 9.980, df = 4, *p* < 0.05) on the nature of costs incurred by the local communities across the villages.

### 3.3. PA-Related Benefits to Local Communities

#### 3.3.1. Claims to Benefit from SVC

Most respondents (*n*=39; 55%) mentioned that they do not get benefits, whereas 45% (*n*=32) of the respondents indicated that they benefit from the SVC. There was no significant difference (KW*χ*^2^ = 1.538, df = 4, *p* > 0.05) on local communities' views in regard to deriving benefits from SVC across the villages.

#### 3.3.2. Nature of Benefits Derived

On the nature of benefits derived from the SVC, fifty percent (50%; *n*=16) of the respondents across the sampled villages claimed that the renovation of local schools is the major benefit derived from SVC, with few respondents (*n*=2; 6%) pointing to borehole drilling as the least derived benefit from the SVC (Table 1). There was no significant difference (KW*χ*^2^ = 6.232, df = 4, *p* > 0.05) on the nature of benefits derived from SVC across the villages.

### 3.4. Attitudes toward SVC

Respondents' attitudes toward SVC based on the following response options: (a) “SVC is more of a liability,” (b) “SVC is for foreign interests,” and (c) “the relationship between SVC and the communities is bad.”

**Figure 5:**
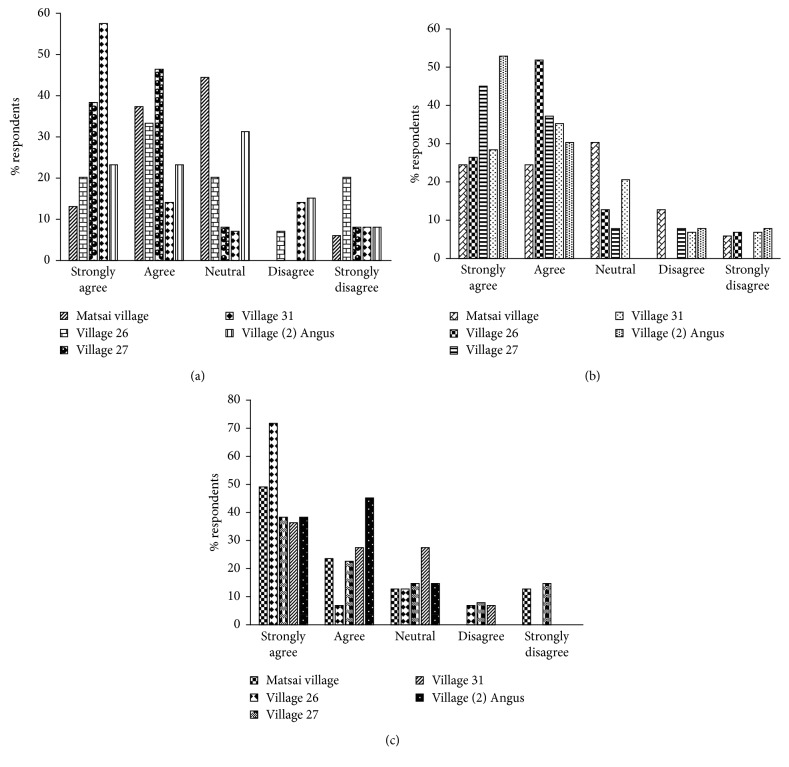
Bar graphs (a)–(c) show percentages of respondents and their attitude ratings towards SVC using the Likert-type scale. Rating scale is as follows: 1 = strongly agree, 2 = agree, 3 = neutral, 4 = disagree, and 5 = strongly disagree. (a) SVC is more of a liability. (b) SVC is for foreign interests. (c) The relationship between SVC and local communities is bad.

- Most (*n*=8; 57%) of the respondents in village thirty-one strongly agreed that SVC is more of a liability, whilst a few (*n*=1; 6%) of the respondents in Matsai village strongly disagreed that SVC is more of a liability. There was no significant difference (KW*χ*^2^ = 5.230, df = 4, *p* > 0.05) on the ratings of respondents attitudes based on (a) (“SVC is more of a liability”), across the villages.
- Many (*n*=7; 54%) of the respondents in village (2) Angus strongly agreed that SVC is for foreign interests, whereas a few (*n*=1; 6%) of the respondents in Matsai village strongly disagreed that SVC is for foreign interests. There was no significant difference (KW*χ*^2^ = 4.359, df = 4, *p* > 0.05) on the ratings of respondents' attitudes based on (b) (“SVC is for foreign interests”), across the villages.
- Majority (*n*=11; 73%) of the respondents in village twenty-six strongly agreed that the relationship between SVC and the communities is bad, whereas a minor proportion (*n*=1; 7%) of the respondents agreed that the relationship between SVC and the communities is bad. There was no significant difference (KW*χ*^2^ = 6.400, df = 4, *p* > 0.05) on the ratings of respondents' attitudes across the villages based on (c) (“the relationship between SVC and the communities is bad”), across the villages, respectively (Figure 5).

### 3.5. Key Informants

#### 3.5.1. Knowledge of Various Laws and Policy Frameworks That Have Provision for Local Communities to Benefit from PAs

Few (*n*=4; 44%) of the key informants had some knowledge of various laws and policy frameworks that could be used to help locals derive conservation benefits from PAs. For instance, they had basic knowledge of the CAMPFIRE-based projects and the wildlife-based land reform policy. The majority (*n*=5; 56%) of the informants had a little idea of any policy-related instruments; the informants reported that the SVC is not conducting awareness campaigns so as to equip local communities with the basic fundamental conservation policy issues. Additionally, all the traditional leaders, villages elders, and ward councilors (*n*=9; 100%) stated that their knowledge of how the SVC community trust a community-based conservation intervention functions is poor.

#### 3.5.2. Challenges Associated with Benefiting from SVC

All traditional leaders, villages elders, and ward councilors (*n*=9; 100%) reported that the devolution of benefits is unclear, whereas the distribution channels are poorly defined. In addition, most of the interviewees, particularly traditional leaders and village elders (*n*=6; 67%), highlighted that the SVC community trust is no longer functional since there are no follow-ups being made to ensure that communities are still benefiting. Furthermore, councilors (*n*=2; 22%) associate the current challenges with the failure of communities to know any policies addressing the roles PAs should play in enhancing local communities well-being. Additionally, all the interviewees (*n*=9; 100%) mentioned that the aspect of resource nationalism through the indigenization of the SVC has resulted in the individual landowners in the SVC to be less concerned with meeting local communities aspirations and needs. The informants believe it is a challenge for their welfare to be addressed as the individual landholders in the SVC are highly antagonistic.

## 4. Discussion

This study provided an opportunity for the first time to examine the nature of costs and benefits of wildlife conservation to local communities living in proximity to SVC. Our results showed that there are five major costs that are being incurred in the study area. The most prevalent costs experienced across the villages were crop raiding and livestock depredation. Costs accruing to local residents can be attributed to security issues and the results are consistent with those of Andersson et al. [16] who observed that encroachment of wild animals into human settlements can be attributed to the state of a PAs security fence. Some sections of SVC's perimeter fence are porous and run down; thus, communities situated at the edge often disproportionately bear the cost of conservation. Crop raiding has impacted on the local communities adversely: as men, women, and children spent most of their time guarding their fields, they have no opportunity to do income-generating activities. Drawing from insights by Mackenzie [28] wildlife-induced costs lead to financial hardships for some households and this has broader implications for development. Since many households make their living solely from subsistence farming, losing crops to wild animals can have serious consequences for food security. The high levels of wildlife-generated costs can be due to the limited number of game scouts involved in the guarding and control of problem animals and the absence of strategies to mitigate human-wildlife conflict. These results concur with those of Shibia [29] at Marsabit National Reserve, Kenya.

In addition, the rate of occurrence of wildlife-induced damage can be due to the fact that the study sites are close to the SVC boundary. Results agree with findings by Salerno et al. [30] and Mackenzie [28] who assert that the distance to the PA-boundary is the primary predictor of conservation losses experienced by local communities. Literature further reveals that costs incurred by edge communities reduce their support for conservation and engender resentment and opposition to it [31, 32]; hence, biodiversity can suffer. In support of this, Gandiwa et al. [11] put forward that illicit wildlife-based activities have been reported to increase with an increase in (i) costs incurred by local communities from wildlife conservation. Local communities are rationale beings and so reciprocity is contingent upon benefits received [33].Thus, the wildlife-induced costs on communities living next to SVC are critical in shaping conservation attitudes and ultimately behaviour. Another factor contributing to the unprecedented levels of wildlife-induced costs could be the marked increase in populations of large herbivores and carnivores, particularly the African elephant (*Loxodonta africana*), spotted hyena (*Crocuta crocuta*), and lions (*Panthera leo*). This can be a major factor as wild game numbers in the SVC are anecdotal. This phenomenon has been also identified by Gandiwa et al. [11] in Gonarezhou National Park.

Furthermore, results on benefits show that a higher proportion of the respondents from the household survey are disgruntled with the nature of benefits derived from the SVC. On a preliminary basis, the dissatisfaction expressed in regard with benefits derived can be due to the awarding of benefits at community level not household level. Other studies have demonstrated that local people hold favourable attitudes toward wildlife conservation when personal benefits are derived from PAs [34–37]. Songorwa [38] also postulate that communities dislike communal benefits; rather, they enjoy them at individual and household levels. This is because most wildlife-induced costs (such as crop raiding and livestock kills) are borne and felt at household level rather than the entire community. Essentially, community benefits undermine people's short-term needs and create a loophole for free riders as they barely address the question of “who pays for, and who benefits from wildlife” [39]. Kideghesho [3] advances that benefits from conservation initiatives targeting the entire community rather than the individual households are condemned as they are termed public goods were the culprits and nonvictims get to enjoy. Also, given the definition of a conservancy, SVC satisfies the definitional parameters on paper but not on the ground; as property holders are highly antagonistic, this can be attributed to the poor flow of benefits to the communities. Depoliticizing conservation issues in the SVC is imperative so as to ensure solidarity among multiple stakeholders which is vital for integrated conservation and development. Also, failure of community leaders in particular and community members in general to recognize laws and policy frameworks authorizing them to profit from conservation outcomes further impedes the derivation of concrete benefits from the SVC.

Moreover, this study recorded that the majority of the respondents across the villages hold negative attitudes toward SVC. Negative attitudes were significantly more common across households that had been affected by wildlife problems. Shibia [29] and Gandiwa et al. [11] indicate that communities have a widespread dislike of and negative attitudes towards most of the common problematic wild animals. Ayivor et al. [31] also argue that anything that threatens a source of livelihood in local people inevitably erodes support for conservation and garners opposition. Therefore, since the majority of the studied households experienced wildlife problems, this justifies the negative conservation behaviour they display toward the SVC. Gillingham and Lee [40] suggest that perceived personal benefits must outweigh perceived disadvantages to engender positive attitudes towards conservation. Furthermore, the negative attitudes are due to the household economic constraints induced by wildlife, as wildlife prey on livestock and the amount of time communities invest in protecting their crops and the livestock. Mackenzie [41] contends that local communities perceived many aspects of wildlife conservation negatively due to costs inflicted by crop raiders and dangerous wild animals around Kibale National Park, Uganda.

## 5. Conclusion and Recommendations

Based on our results, it can be concluded that costs outweigh benefits in local communities living adjacent to SVC and that there are no formal benefit sharing mechanisms (BMSs), leading to local communities having negative attitudes towards SVC and wildlife conservation in general. It is recommended that costs incurred by local communities can be offset by a number of actions such as (i) putting in place formalized BSMs to ensure a more consistent flow of benefits to local people living on the edge. This is critical as Jensen [42] argues that the economic man never performs without incentives (no benefits no conservation); (ii) deliberate affirmative action where locals should be employed as a form of benefit of living close to a PA; (iii) there is need to document the economic, social, and opportunity costs of SVC on local communities thus creating inventories. These inventories can support the development of conservation strategies to minimize the burden of SVC on edge communities while sustainably managing biodiversity; (iv) training local residents to promote tourism activities, this may include training them to make arts and crafts that can be sold to tourists. This will earn income for the local people and improve their livelihoods thereby creating a more favourable climate for conservation. The SVC management should help to secure market for these products; and (v) holistically, SVC authorities need to have the capacity to embark on regular outreach programmes to dialogue with community members and to listen to their concerns. Regular dialogue will help induce pro-conservation attitudes, reduce acrimony, and curtail conflict situations.

## Figures and Tables

**Table 1:** Responses toward the nature of benefits derived from SVC across the villages.

Village (*s*)	Bridge construction	School construction	Protein supplementation	Borehole drilling	Road maintenance
Matsai	3	1	2	0	0
26	0	3	2	1	0
27	1	4	0	1	1
31	2	3	0	0	0
Village (2) Angus	1	5	0	0	2
Total (*n*=32)	7 (22%)	16 (50%)	4 (13%)	2 (6%)	3 (9%)

## Data Availability

The data used to support the findings of this study are available from the corresponding author upon request.
